# Harnessing Cellular Immunotherapy for EBV‐Associated Malignancies: Current Advances and Future Directions

**DOI:** 10.1111/jcmm.70603

**Published:** 2025-05-22

**Authors:** Yang Gao, Di Wang, Chunrui Li

**Affiliations:** ^1^ Department of Hematology, Tongji Medical College, Tongji Hospital Huazhong University of Science and Technology Wuhan Hubei China

**Keywords:** cytotoxic T lymphocyte, Epstein–Barr virus, nasopharyngeal carcinoma, post‐transplant lymphoproliferative disorder, T‐cell receptor

## Abstract

Standard treatments for EBV‐associated malignancies, such as chemotherapy and radiotherapy, demonstrate limited efficacy in relapsed or refractory cases, underscoring an urgent need for innovative therapeutic strategies. Recent advances in immunotherapy—particularly EBV‐specific cytotoxic T lymphocytes and dendritic cell vaccines—have shown promise for both treatment and prevention. Engineered T cell therapies, including T‐cell receptor (TCR) and chimeric antigen receptor (CAR) approaches targeting EBV antigens such as LMP1 and gp350, are progressing in clinical development. Compared to conventional intensive therapies, which often require prolonged administration and are associated with significant toxicity, cellular immunotherapy offers a favourable safety profile alongside robust in vivo T cell expansion and potent antitumor effects. Although preclinical and clinical trial results are encouraging, further refinement of therapeutic protocols is critical to enhance efficacy and improve access for diverse patient populations. In this review, we summarise the rationale for EBV‐directed cellular therapies, outline their clinical applications to date, and discuss current limitations as well as emerging opportunities to optimise these strategies.

## 1 Introduction

Epstein–Barr virus (EBV), the first oncogenic virus identified in humans, is also known as human herpesvirus 4 (HHV‐4) [1]. EBV has a characteristic three‐layered structure: the outer lipid bilayer envelope containing viral glycoproteins that facilitate host recognition and membrane fusion, the inner layer is a pseudo‐icosahedral nucleocapsid that encloses the 172‐kb double‐stranded linear DNA genome, and the intermediate polymorphic periplasmic compartments filled with viral proteins [2].

Approximately 95% of the global population is infected with EBV, with most individuals experiencing lifelong asymptomatic infections characterised by undetectable viral replication [3]. Detectable EBV infections, however, often manifest as infectious mononucleosis (IM) in adolescents and young adults, presenting with symptoms such as fever, malaise, sore throat, and lymphadenopathy [4]. Both intrinsic factors (e.g., genetic mutations and deficiencies) and extrinsic factors (e.g., immunosuppression, salted or preserved diet) can lead to the development of EBV‐associated lymphoma and epithelial cancers by multiple ways like modulating host innate and adaptive immune responses, inhibiting apoptotic and differentiation pathways, and affecting oncogene expression [5].

Despite favourable clinical outcomes in EBV‐associated malignancies after conventional chemotherapy or radiotherapy, a substantial proportion of patients remain refractory to these treatments. Moreover, standard treatment modalities present several challenges, including treatment‐related complications, as well as the risk of relapse [6]. The secretion of EBV‐specific targetable antigens, along with the predominant anti‐infective activity of immune cells, suggest that cellular‐based immunotherapies may represent a viable and potentially more effective therapeutic strategy for EBV‐associated malignancies [7]. Therefore, understanding the infection and immune processes of EBV is crucial for advancing current cellular immunotherapies. Based on this, we will outline the mechanisms and advantages of current EBV‐associated cellular‐based immunotherapies, as well as the potential challenges they may face in clinical application (Table 1).

**TABLE 1:** A brief summary of cellular‐based immunotherapies for EBV‐associated malignancies.

Therapy	Mainly targeted molecules	Advantages	Limitations
CTL [8, 9]	– EBV specific antigens like EBNA3A‐3C and LMP	– Established methodology and widely used – Safe and effective therapeutic or preventive effects	– Strict condition and long‐time cost of preparation – Unstable persistence of CTLs – Lack of standardised treatment management like infusion timing and dosage – Immune evasion mediated by EBV
TCR‐T cell [10, 11]	– EBV specific antigens like LMP and EBNA1	– Ultra‐sensitive recognition of low‐level variation of intracellular antigens – Enhanced specificity and sensitivity – Rapidly and efficiently generate large quantities of EBV‐specific T cells	– HLA‐restricted and high‐cost – Toxicity effects or functional impairment of TCR‐Ts from unstable TCR expression or mismatch – Immune evasion mediated by EBV – Lack of sufficient clinical trials to assess its efficacy and safety
CAR‐T cell [12, 13]	– Membrane surface molecules like gp350 and EBV specific antigens like LMP	– A wider array of tumour‐associated antigens – Enhanced specificity and sensitivity	– High cost and complexity in preparation – Toxicity effects like CRS and host rejection response – Immune evasion mediated by EBV – Lack of sufficient clinical trials to assess its efficacy and safety
DC vaccine [14, 15]	– EBV specific antigens and unspecific viral antigens	– Enhancement of both innate and adaptive immune responses – Capacity of targeting multiple antigen epitopes – High tolerance and safety	– Lack of efficient methodologies for the isolation and generation of DCs – Limited therapeutic efficacy resulting from immune evasion and suboptimal T‐cell immune responses induced by DC vaccines

Abbreviations: CAR, chimeric antigen receptor; CRS, cytokine release syndrome; CTL, cytotoxic T lymphocyte; DC, dendritic cell; EBNA, Epstein–Barr nuclear antigen; EBV, Epstein–Barr virus; Gp350, glycoprotein 350; LMP, latent membrane protein; TCR, T‐cell receptor.

## 2 Infection Mechanisms and Immune Response in EBV Infection

After infecting the host, EBV establishes two distinct phases of infection: the latent phase and the lytic phase. EBV is primarily transmitted through saliva, initiating infection in the oropharynx and tonsils before establishing latency in sIgD+ naïve B lymphocytes that migrate to the oral cavity [16]. During latency, the virus exploits host cellular machinery for replication, minimising viral gene expression to evade immune detection. EBV‐associated cancers are stratified based on their latent gene expression profiles (Table 2) [17].

**TABLE 2:** Latency phase and gene expression of EBV‐associated cancers.

Latency phase	MicroRNA	EBNA	LMP	EBV‐associated diseases
0	– EBER – BART	/	/	– Healthy carriers
I	– EBER – BART	– EBNA1	/	– B lymphoblastic leukaemia – Burkitt's lymphoma – Gastric cancer
II	– EBER – BART	– EBNA1	– LMP1 – LMP2	– Nasopharyngeal carcinoma – Hodgkin's lymphoma – Nasal T/natural killer‐cell lymphomas – Diffuse large B cell lymphoma
III	– EBER – BART – BHRF1	– EBNA1 – EBNA2 – EBNA3A‐3C – EBNA4	– LMP1 – LMP2	– Post‐transplant lymphoproliferative disorders – AIDS‐associated non‐Hodgkin's lymphoma – Diffuse large B cell lymphoma

Abbreviations: AIDS, acquired immunodeficiency syndrome; BART, BamHI‐a rightward transcript; BHRF1, BamHI‐H rightward frame 1; EBER, Epstein–Barr virus‐encoded RNA; EBNA, Epstein–Barr nuclear antigen; LMP, latent membrane protein.

The transition from latency to the lytic phase, commonly triggered by plasma cell differentiation, leads to active viral replication and immune evasion. This process involves mechanisms such as the production of IL‐10, which constitutively diminishes the efficacy of the CTL response [18, 19]. During the lytic phase, replicated viral genomes are packaged into pre‐formed capsid particles and released through exocytosis. The intercellular transport facilitates the secretion of EBV particles into saliva, enabling efficient host‐to‐host transmission [20].

EBV infection activates both innate and adaptive immune responses for pathogen clearance and tumour cell elimination. Activated immune cells, particularly cytotoxic T lymphocytes (CTLs), are central to recognising and eliminating EBV‐infected or malignant cells. For example, cytotoxic CD4+ T lymphocytes mediate viral clearance via perforin release and the Fas/FasL pathway [21, 22]. Cytotoxic CD8+ T lymphocytes induce EBV‐infected cells apoptosis via secretion of IFN‐γ, granzymes, and perforins [23]. These robust immune responses underline the potential of harnessing cellular immunity to control and eliminate EBV‐associated malignancies effectively.

## 3 Adoptive EBV‐Specific CTL Therapy

Adoptive EBV‐CTL therapy enhances immune capacity by generating and transferring T cells specifically targeting EBV antigens. Systemically produced EBV‐CTLs consist of both CD8+ and CD4+ T lymphocytes. CD8+ T cells dominate the elimination of infected or tumour cells due to their immunogenic responses to EBV antigens, particularly from latent cycle proteins like EBNA3A, 3B, and 3C. Meanwhile, subdominant responses arise from LMP2, with EBNA2 and LMP1 being less frequent [24]. CD4+ T cells play a supportive role by secreting cytokines, enhancing overall immune response [25, 26].

Various strategies are currently employed to produce EBV‐CTLs from patient‐derived peripheral blood mononuclear cells (PBMCs), including the use of EBV‐transformed B lymphoblastoid cell lines (B‐LCLs) or DCs for CTL preparation [27, 28]. For post‐transplant lymphoproliferative disorder (PTLD) following allogeneic haematopoietic stem cell transplantation (allo‐HSCT), PBMCs from EBV‐positive donors can be stimulated with peptide mixtures to isolate EBV‐CTLs using IFN‐γ capture for urgent use [29]. The most widely used source of EBV‐CTLs is third‐party donor libraries. These “off‐the‐shelf” CTLs are cryopreserved and selected based on partial HLA matching, enabling immediate availability and broader application [30]. A summary of clinical studies on EBV‐CTLs (more than 5 participants) for the treatment of EBV‐associated cancers, published since 2010, is presented in Table 3.

**TABLE 3:** Clinical evidence for adoptive transfer of EBV‐CTLs for EBV‐associated diseases from 2010.

First author (year)	Types of patients	No. of patients	Preparation of CTLs	Treatment or preventive effects	Adverse events related to EBV‐CTLs
Heslop (2010) [27]	Proven or probable PTLD	114	EBV‐transformed B‐LCLs simulated CTLs	– Prophylaxis group (101 patients): PTLD incidence 0% – Treatment group (13 patients): CR 84.6%	– Grade 3 GVHD (1 patient) – Grade 3 swelling or sloughing (2 patients) – Grade 3 pleural effusion (1 patient)
Moosmann (2010) [29]	R/R PTLD	6	IFN‐γ capture	– CR 50%	– Grade 3 aggravated facial edema requiring airway protection (1 patient)
Louis (2010) [31]	R/R NPC	23	EBV‐transformed B‐LCLs simulated CTLs	– Active disease (15 patients): CR 33.3%; PR 13.3%; SD 20% – Cohort in remission (8 patients): CR 62.5%	– No treatment‐related toxicities observed
Secondino (2011) [32]	R/R NPC	11	EBV‐transformed B‐LCLs simulated CTLs	– PR 18.2%; MR 9.1%; SD 27.3%	– Grade 3 swelling requiring anti‐inflammatory therapy (1 patient) – Grade 3 neutropenia (4 patients) – Grade 3 fever and tremors requiring antihistamine therapy (1 patient)
Doubrovina (2011) [33]	PTLD	19	EBV‐transformed B‐LCLs simulated CTLs	– CR 68.4%	– No grade 3 or higher toxicities observed
Smith (2012) [28]	R/M NPC	14	AdE1‐LMPpoly‐transduced PBMCs induced LMP‐CTLs	– SD 71.4%	– Grade 3 epistaxis (1 patient)
Icheva (2012) [34]	EBV viremia or PTLD	10	IFN‐γ capture	– CR 30%	– Grade 4 GVHD (2 patients)
Bollard (2013) [35]	Proven or probable relapse HL or NHL	50	AdV‐LMPpoly‐transduced DCs induced LMP‐CTLs	– Active disease (21 patients): CR 52.4%; PR 9.5% – Cohort in remission (29 patients): CR 93.1%	– Grade 3 CNS deterioration (1 patient) – Grade 3 respiratory complications (1 patient)
Chia (2013) [36]	R/M NPC after GC chemoth‐erapy	35	EBV‐transformed B‐LCLs simulated CTLs	– CR 5.7%; PR 31.7%; SD 20%	– No grade 3 or higher toxicities observed
Vickers (2014) [30]	PTLD	11	Third‐party EBV‐specific CTL library	– CR 72.7%; PR 9.1%	– Grade 3 GVHD (1 patient)
Cho (2015) [37]	ENKTCL in initial remission	10	LMP1 or LMP2a RNA‐transduced DCs induced LMP‐CTLs	– CR 90%	– No treatment‐related toxicities
Huang (2017) [38]	R/M NPC	21	EBV‐transformed B‐LCLs simulated CTLs	– CR 4.8%; SD 9.6%	– Grade 5 sepsis and pulmonary insufficiency (1 patient)
Smith (2017) [39]	NPC	29	AdE1‐LMPpoly‐transduced PBMCs induced LMP‐CTLs	– Active disease (20 patients): SD 60% – Cohort in remission (9 patients): CR 66.7%	– No grade 3 or higher toxicities observed
Chiou (2017) [40]	PTLD	10	Third‐party EBV‐specific CTL library	– CR 70%; PR 10%	– No grade 3 or higher toxicities observed
Bollard (2018) [41]	Proven or probable relapse HL	8	Ad5f35d‐LMPpoly‐transduced DCs induced LMP‐CTLs	– Active disease (7 patients): CR 28.6%; PR 14.3%; SD 57.1% – Cohort in remission (1 patient): CR 100%	– No treatment‐related toxicities
McLaughlin (2018) [42]	R/R B‐ or NK/T‐cell EBV‐associated lymphoma or LPD	26	Ad5f35d‐LMPpoly‐transduced DCs induced LMP‐CTLs	– Active disease (7 patients): PR 28%; SD 14% – Cohort in remission (19 patients): CR 78.9%	– Grade 4 hepatic necrosis (1 patient)
Prockop (2018) [43]	R/R PTLD	46	Third‐party EBV‐specific CTL library	– The HCT group (33 patients): CR 57.6%; PR 9.1% – The SOT group (13 patients): CR 15.4%; PR 38.5%	– No grade 3 or higher toxicities observed
Wistingha‐usen (2023) [44]	CD20+ PTLD	15	Third‐party EBV‐specific CTL library	– R/R PTLD group (5 patients): SD 20% – PTLD group (10 patients): CR 40%; PR 30%	– Grade 3 CRS (1 patient)
Nikiforow (2024) [45]	R/R PTLD	26	Third‐party EBV‐specific CTL library	– The HCT group (14 patients): CR 28.6%; PR 21.4% – The SOT group (12 patients): CR 50%; PR 33.3%	– Grade 3 or higher toxicities including abdominal pain, colitis, GVHD and pneumonitis observed (3 patients)
Toh (2024) [8]	R/M NPC	164	EBV‐transformed B‐LCLs simulated CTLs	– CR 3.7%; PR 56.7%; SD 28%	– Grade 3 anaemia and leukopenia (1 patient)

Abbreviations: Ad5f35, adenovirus serotype 5/serotype 35 hybrid virus; AdE1, adenovirus E1; AdV, adenovirus; AE, adverse event; CR, complete response; CRS, cytokine release syndrome; CTL, cytotoxic T lymphocyte; DC, dendritic cell; ENKTCL, extranodal natural killer/T‐cell lymphoma; GC, gemcitabine‐carboplatin; GVHD, graft‐versus‐host disease; HL, Hodgkin's lymphoma; HCT, haematopoietic stem cell transplantation; LCL, lymphoblastoid cell line; LMP, latent membrane protein; LPD, lymphoproliferative disorder; NHL, non‐Hodgkin's lymphoma; No., number; NPC, nasopharyngeal carcinoma; PR, partial response; PTLD, post‐transplantation lymphoproliferative disorder; R/M, relapsed or metastatic; R/R, relapsed or refractory; SD, stable disease; SOT, solid organ transplantation.

### 3.1 Applications in EBV‐PTLD

The first CTL therapies targeted early lytic and immunodominant latent antigens like EBNA3 proteins, which are expressed in type III latency malignancies such as PTLD. PTLD, a group of highly fatal disorders, arises from T cell suppression after solid organ transplantation (SOT) or haematopoietic stem cell transplantation (HSCT) [46]. Traditional PTLD treatments, including immunosuppression reduction, chemotherapy, and rituximab, have limited success. Median survival is only 0.7 months for HCT recipients and 4.1 months for SOT recipients with refractory disease, highlighting the urgent need for better therapies [47, 48].

Tabelecleucel, the first third‐party EBV‐specific CTL therapy approved in the European Union, demonstrated a 65% overall response rate in relapsed/refractory (R/R) PTLD, with sustained survival benefits and minimal adverse events [45]. Similarly, in a 15‐year follow‐up of prophylactic CTLs post‐HSCT, none of the 90 treated patients developed PTLD compared to an 11% incidence in untreated individuals [27].

### 3.2 Efficacy in NPC and Lymphomas

EBV‐CTLs targeting type II latency antigens (e.g., EBNA1, LMP1, LMP2) show promise for nasopharyngeal carcinoma (NPC) and lymphomas. Non‐keratinizing NPC, strongly associated with EBV, often metastasizes, leading to poor outcomes [49]. A phase II trial combining gemcitabine‐carboplatin (GC) chemotherapy and EBV‐CTLs as first‐line treatment for recurrent/metastatic (R/M) NPC resulted in superior median survival (26.6 months) compared to standard platinum‐based chemotherapy (10.6 months) [36]. Despite favourable safety and activity profiles, a randomised phase III trial of GC with EBV‐CTLs failed to show significant survival improvements over GC alone [8].

EBV‐associated Hodgkin lymphoma (HL) and non‐Hodgkin lymphoma (NHL), often linked to type II latency, also benefit from CTL therapies. HL patients typically respond well to combined chemotherapy and radiotherapy, achieving cure rates exceeding 80%. However, R/R HL cases have limited options, with high‐dose chemotherapy offering only 50% 5‐year survival [50]. Advanced techniques, including adenoviral vector‐based T cell expansion, generate CTLs targeting antigens like LMP1, LMP2, and EBNA1. In clinical trials, these CTLs achieved sustained remission in relapsed HL/NHL, demonstrating excellent safety and efficacy [51].

### 3.3 Challenges and Future Directions

Despite favourable tolerance and significant progress, particularly in the treatment of EBV‐PTLD, EBV‐CTL therapy faces hurdles. In some clinical studies of EBV latent phase II diseases, such as R/M NPC, EBV‐CTLs have shown limited efficacy, with no significant improvement in OS. The limited therapeutic efficacy of EBV‐CTLs may be linked to the resource and time intensive nature of CTL generation, delaying treatment and potentially excluding patients with rapidly progressing disease [8]. Additionally, the weaker immunogenicity of EBV‐CTLs in targeting EBV latent phase II antigens may further contribute to the suboptimal outcomes. Third‐party CTL libraries address the issue of extended preparation times, thereby shortening the waiting period for treatment and potentially minimising the progression of the disease. However, third‐party T cells are predominantly applicable to EBV‐associated PTLD, with their persistence generally being limited, necessitating multiple infusions to attain optimal therapeutic efficacy. The exact predictive factors for treatment efficacy remain unclear, although it is known that factors such as dose, T cell immunophenotype, and HLA matching do not appear to correlate with therapeutic response [43].

Host factors, including alloreactive antibodies and residual alloreactive T cells, may contribute to the rejection of EBV‐CTLs after infusion, compromising the cellular activity and persistence. Furthermore, EBV has developed mechanisms to evade EBV‐CTLs, ranging from epitope variation between different EBV strains to viral‐encoded evasion proteins. These proteins can hijack antigen processing and presentation pathways, downregulate specific HLA alleles, or directly suppress T cell function [9]. For instance, researchers have shown that EBV‐encoded BART cluster miRNAs promote T‐cell apoptosis and immunosuppression by upregulating PD‐L1 expression in NPC and gastric carcinoma (GC) cells, facilitating tumour immune evasion in xenograft mouse models [52]. Furthermore, studies have demonstrated that PD‐1 is upregulated on EBV‐LMP2A‐specific CD8+ T cells and is associated with impaired CTLs function. Genetic disruption of PD‐1 in EBV‐LMP2A‐induced CTLs was shown to enhance their cellular immune response and cytotoxic activity against EBV‐positive GC cell lines. In a xenograft mouse model of EBV‐associated GC, these PD‐1‐deficient T cells significantly improved local tumour control and prolonged survival when administered in combination with low‐dose radiotherapy [53]. These findings suggest that targeting the PD‐1/PD‐L1 axis through genetic disruption or immune checkpoint blockade could represent a promising strategy to enhance the efficacy of adoptive T cell therapies. Several other factors may influence the efficacy, including the distinct technical approaches used in the generation of EBV‐CTLs, the heterogeneity of patient populations at different stages of disease, the effects of prior treatments, the presence of comorbid conditions and genetic factors that predispose individuals [38].

Future strategies to address the limitations of EBV‐CTL therapy include the development of engineered CTLs, as well as their combination with chemotherapy agents, histone deacetylase inhibitors or proteasome inhibitors, to enhance the immunogenic expression of EBV antigens in tumour cells. Lymphodepletion prior to T‐cell infusion may help to enhance the expansion and persistence of infused cells, making it a potentially valuable approach to improve the effectiveness of CTL therapies [31]. In addition, the variability in clinical outcomes underscores the critical need to further investigate patient‐specific immune factors and treatment‐related variables, such as prior therapies and comorbidities, to predict and optimise therapeutic efficacy. Furthermore, determining the optimal timing for EBV‐CTL infusion is essential for maximising treatment outcomes.

## 4 EBV‐Specific TCR‐T Cell Therapy

While CTL therapy offers significant benefits, limitations such as immune evasion underscore the necessity for more advanced strategies, including TCR and CAR engineered T cells [54]. TCRs are heterodimeric proteins composed primarily of α and β chains, with a smaller subset formed by γ and δ chains. The αβ TCR lacks intrinsic signalling capabilities and relies on the CD3 complex and zeta (ζ) chains to transduce activation signals upon engagement with peptide–MHC complexes. This signalling cascade drives T cell proliferation, differentiation, and the establishment of memory T cells, enabling robust immune responses against pathogens and tumours upon re‐exposure [55].

**FIGURE 1:**
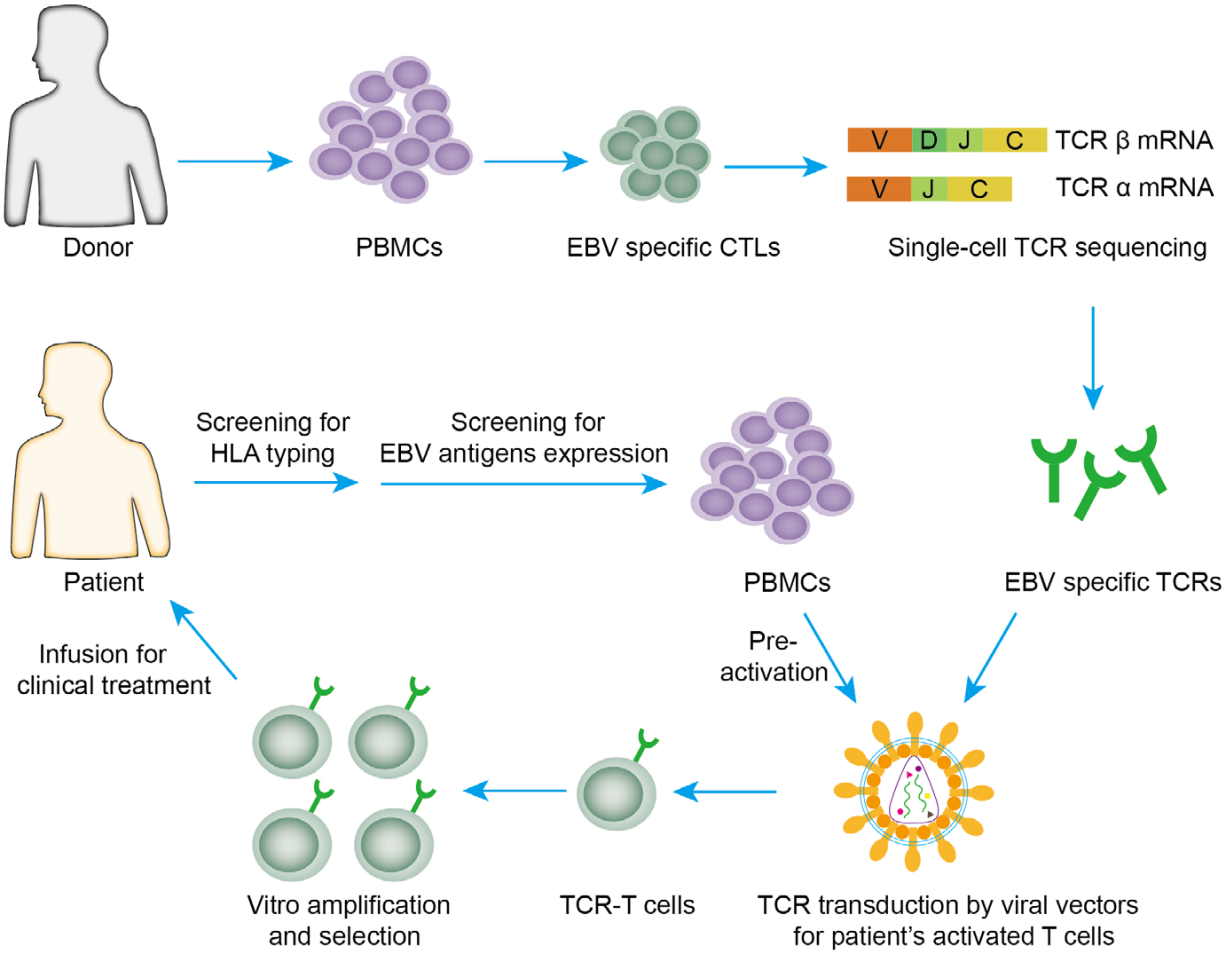
Flowchart for preparation process of EBV antigen‐reactive TCR‐T cells. PBMCs are isolated from healthy volunteers and used to generate EBV‐specific T cells under good manufacturing practice conditions. The TCR α/β chains were synthesised and cloned into a viral vector. Patients are initially screened for HLA typing and EBV antigen positivity. Patients eligible for TCR‐based immunotherapy will have their PBMCs isolated and pre‐activated, followed by the introduction of specific TCRs into activated peripheral T cells via viral vectors to generate EBV antigen‐reactive TCR‐Ts. The successfully transduced T cells, endowed with specific recognition and cytotoxic capabilities, are subsequently selected, expanded ex vivo, and further utilised for clinical therapeutic research. CTL, cytotoxic T lymphocyte; PBMC, peripheral blood mononuclear cell; HLA, human leukocyte antigen; TCR, T‐cell receptor.

Engineered T cells, modified with HLA‐matched EBV‐specific TCRs, offer a promising immunotherapeutic approach for EBV‐associated malignancies (Figure 1). These cells selectively recognise and eliminate EBV‐infected cells, enhancing therapeutic efficacy [54]. A summary of clinical trials on EBV‐TCR therapies conducted since 2010 is presented in Table 4.

**TABLE 4:** Clinical trials of EBV TCR‐T cells for EBV‐associated diseases from 2010.

NCT number	Status	Phase	Conditions	Interventions	Primary outcome measures
NCT03648697	Unknown	Phase 2	R/M NPC	TCR‐T cells	– Number of participants with AEs in 60 days after treatment
NCT04139057	Recruiting	Phase 1/2	NHSCC	TCR‐T cells with a PD1 antagonist	– MTD of TCR‐T cells in 8 weeks after treatment
NCT03925896	Unknown	Phase 1	R/M NPC	LMP2‐specific TCR‐T cells	– MTD of TCR T‐cell therapy in 2 years after treatment
NCT04156217	Completed	Phase 1	EBV Emia or PTLD	TCR‐T cells	– AEs and percentage of participants with AEs in 3 months after treatment
NCT05587543	Recruiting	Phase 1	R/R NPC	TCR‐T cells or CAR‐T cells	– DLT in 1 year after treatment – MTD of TCR‐T cells and CAR‐T cells – Incidence of AE, SAE, AESI in 1 year after treatment
NCT04509726	Recruiting	Phase 1/2	R/M NPC	TCR‐T cells with cytokine auto‐secreting element	– MTD of TCR‐T cells in 8 weeks after treatment
NCT06135922	Recruiting	Phase 1	EBV infection or HLH	TCR‐T cells	– AEs and percentage of participants with AEs in 1 year after treatment
NCT06119256	Recruiting	Phase 1	EBV infection after allo‐HSCT	TCR‐T cells	– AEs and percentage of participants with AEs in 1 year after treatment

Abbreviations: AE, adverse event; AESI, adverse events of special interest; allo‐HSCT, allogeneic haematopoietic stem cell transplantation; CAR, chimeric antigen receptor; DLT, dose‐limiting toxicity; HLH, hemophagocytic lymph histiocytosis; LMP, latent membrane protein; MTD, maximum tolerated dose; NHSCC, neck and head squamous cell carcinoma; NPC, nasopharyngeal carcinoma; PD‐1, programmed cell death protein 1; PTLD, post‐transplantation lymphoproliferative disorder; R/M, relapsed or metastatic; R/R, relapsed or refractory; SAE, serious adverse event; TCR, T‐cell receptor.

### 4.1 MP Antigens as Therapeutic Targets

Among EBV‐associated antigens, latent membrane proteins (LMPs) rank as the third most critical in cancer immunotherapy due to their high oncogenic potential and specificity [56]. LMP1, a 386‐amino acid protein, facilitates B cell immortalization by activating DNA synthesis and NF‐κB signalling, thereby promoting proliferation and resistance to apoptosis through upregulation of Bcl‐2 and Bfl‐1 [57, 58, 59].

Recent studies demonstrated the efficacy of LMP1‐specific TCRs in targeting LMP1‐overexpressing tumour cells. In murine models, adoptive transfer of LMP1‐TCR‐engineered T cells delayed tumour progression and extended survival, overcoming challenges like low availability of LMP1‐CTLs and high LMP1‐associated cytotoxicity [60].

LMP2A, characterised by its immunoreceptor tyrosine‐based activation motif (ITAM), interferes with normal B‐cell receptor signalling, promoting B cell transformation [61]. LMP2A‐specific TCR‐T cells exhibit robust anti‐tumour activity, including IFN‐γ production and inhibition of tumour progression in preclinical models [62]. A specific HLA‐A*1101‐restricted TCR offers broad therapeutic potential for NPC, where 40% of patients express this allele. These TCR‐engineered T cells have demonstrated efficacy in antigen‐specific responses and tumour control in both preclinical and patient‐derived models [10].

### 4.2 EBNA1 Antigen as a Target in NPC

The EBNA1 protein is essential for both viral replication and oncogenesis. EBNA1 binds to the cis‐acting element oriP, which serves as the origin of replication, triggering the initiation of EBV DNA replication. Additionally, EBNA1 enhances the transcription of viral genes and can substitute for ubiquitin‐specific protease 7 (USP7) by binding to p53, preventing p53‐mediated apoptosis and promoting infected cellular survival [63, 64]. However, HLA‐DP‐restricted CD4+ TCRs targeting EBNA1 have shown promise. In NPC xenografts, TCR‐engineered CD4+ T cells effectively killed EBNA1‐expressing tumours while activating CD8+ T cells for a synergistic anti‐tumour response [65].

### 4.3 Challenges and Future Directions

TCR therapy offers notable advantages over EBV‐CTL therapy, particularly in its capacity to rapidly and efficiently produce large quantities of EBV‐specific T cells, independent of the patient's prior immune status [10]. Primarily, it is constrained by strict HLA allele compatibility, limiting its applicability to patients with specific HLA subtypes and corresponding epitopes. Additionally, TCR‐T therapy requires intact antigen presentation functionality, which can be compromised by EBV‐mediated immune evasion. This immune evasion is attributed to several factors, including EBV‐mediated downregulation of HLA expression, loss of antigenic epitopes, the presence of immunosuppressive factors, and the modulatory effects of the tumour microenvironment [10, 66, 67].

Suppressive immune cells and molecules in the tumour microenvironment, including M2 macrophages and matrix metalloproteinase 9 (MMP9), can impair the function of TCR‐T cells, while chronic antigen exposure may induce T cell exhaustion. Moreover, apoptotic tumour cells killed by TCR‐T cells can release chemokines that recruit immune suppressor cells, further inhibiting the anti‐tumour response [66]. What's more, the integration of endogenous and newly introduced TCR chains can result in TCRs with undefined specificity, which may cause the development of self‐reactive T cells and off‐target toxicity [11].

Isolating tumour‐specific αβ TCR T cells from donors or patients via in vitro stimulation with peptides or antigens is often inefficient, due to low T cell frequencies and the lack of robust expansion protocols. Ongoing researches seek to develop a procedure for generating individualised EBV‐specific TCR‐Ts. A pilot study has shown the feasibility and effectiveness of using LMP2A‐encoding lentivectors to generate LMP2A‐overexpressing LCLs for screening LMP2A‐reactive TCRs in EBV‐associated malignancies. This approach could also be extended to identify neoantigen‐specific TCRs in other solid tumours [62]. Epitope‐loss EBV variants can be avoided by using multiple TCRs targeting additional epitopes in EBV specific antigens, although some of which are again restricted through HLA alleles [10]. Current studies are dedicated to optimising TCR design to improve antigen specificity and minimise associated risks. Emerging strategies, such as single‐chain TCR constructs, aim to enhance both the safety and efficacy of TCR‐based therapies. However, unstable TCR surface expression persists, and only a fraction of T cells with the introduced single TCR chain are able to bind the peptide–MHC tetramer [68].

Combining TCR‐T therapy with enhanced co‐stimulatory molecules, such as adhesion and accessory molecules, or modifying the tumour microenvironment (e.g., using MMP9 inhibitors), could potentially improve therapeutic efficacy [66, 69]. These strategies hold promise for advancing the clinical application of TCR‐engineered T cell therapy.

## 5 EBV‐Specific CAR‐T Cell Therapy

In addition to TCR‐T cells, CAR‐T cells are also a key focus of engineered T cell therapy research. A typical CAR consists of several essential components: an extracellular antigen‐binding domain, typically a single‐chain variable fragment (scFv) derived from a specific antibody, a flexible hinge region, a transmembrane domain, a co‐stimulatory domain, and the signalling domains of the TCR CD3ζ chain. This co‐stimulatory domain facilitates T‐cell activation similarly to endogenous TCR signalling [70, 71].

Compared to TCR‐T cells, CARs are designed to directly recognise and bind to surface‐accessible or secreted antigens, bypassing the limitations of MHC‐dependent antigen presentation. This enables CARs to target a broader range of biomolecules, extending beyond the proteome to include glycoproteins and other macromolecules. These glycan structures, often aberrantly expressed on tumour cells, exhibit significant differences compared to their normal counterparts, enabling CAR‐T cells to more effectively distinguish between malignant and healthy tissues [13]. A summary of clinical trials on EBV‐CAR therapies since 2010 is presented in Table 5.

**TABLE 5:** Clinical trials of EBV CAR‐T cells for EBV‐associated diseases from 2010.

NCT number	Status	Phase	Conditions	Interventions	Primary outcome measures
NCT02980315	Unknown	Phase 1/2	NPC	LMP1 CAR‐T cells	– All cause mortality in 1 year after treatment
NCT04288726	Recruiting	Phase 1	R/R CD30‐positive lymphomas	CD30.CAR‐EBVST cells	– DLT rate of CAR‐T cells in 28 days after treatment
NCT05654077	Recruiting	Phase 1	R/R NPC	CAR‐T cells with FC	– DLT of CAR‐T cells in 28 days after treatment
NCT05587543	Recruiting	Phase 1	R/R NPC	TCR‐T cells or CAR‐T cells	– DLT in 1 year after treatment – MTD of TCR‐T cells and CAR‐T cells – Incidence of AE, SAE, AESI in 1 year after treatment
NCT06176690	Not yet recruiting	Phase 1	R/R CD30‐positive lymphomas	C7R.CD30.CAR‐EBVSTs	– DLT rate of CAR‐T cells in 28 days after treatment

Abbreviations: AE, adverse event; AESI, adverse events of special interest; C7R, constitutive interleukin‐7 receptor; CAR, chimeric antigen receptor; DLT, dose‐limiting toxicity; EBVST, Epstein–Barr virus‐specific T lymphocyte; FC, fludarabine‐cyclophosphamide; LMP, latent membrane protein; MTD, maximum tolerated dose; NPC, nasopharyngeal carcinoma; R/R, relapsed or refractory; SAE, serious adverse event; TCR, T‐cell receptor.

### 5.1 Potential Targets of CAR‐T Therapy

The EBV envelope glycoprotein gp350 is a primary focus for CAR‐T therapy. The gp350 antigen, highly conserved across all EBV subtypes, is predominantly expressed during viral lysis and reactivation of infected host cells, while being sporadically present on the surface of latently infected cells [72]. Researchers have developed and evaluated 7A1mAb‐gp350CAR‐T cells as both preventive and therapeutic strategies to combat EBV lytic reactivation and associated malignancies. In vitro experiments demonstrated robust IFN‐γ production, proliferation, and cytotoxicity by CAR‐T cells co‐cultured with EBV latently infected B95‐8 cells. In humanised mouse models, these CAR‐T cells reduced lymphoproliferation, inhibited malignancy progression, and mitigated inflammation [73].

Encouraged by these preclinical successes, researchers generated gp350CAR‐T cells using lentiviral vectors. These CAR‐T cells demonstrated potent cytotoxicity against gp350+ cell lines derived from lymphoma, NPC, and GC. In vivo studies further confirmed their dose‐dependent tumour inhibition effects in NPC models, correlating with detectable CAR‐T cell persistence in various tissues and enhanced tumour infiltration [74].

In addition to gp350, several other antigens are being explored for EBV‐specific CAR‐T cell therapy. LMP1‐specific CAR‐T cells have been developed and have demonstrated effective cytotoxicity against LMP1‐positive target cells in vitro, as well as inhibition of LMP1‐expressing NPC tumour growth in subcutaneous xenograft mouse models [75]. A third‐generation CAR‐T cell incorporating a CD137 signalling domain has shown enhanced immune responses in both in vitro and in vivo studies. Given that CD137 supports T‐cell proliferation and survival, HELA/137CAR‐T cells exhibited prolonged persistence in mouse models, effectively addressing the common limitation of short‐lived efficacy observed in earlier CAR‐T therapies [71].

### 5.2 Challenges and Future Directions

Aside from the drawbacks of manufacturing complexity and cost, CAR‐T therapies also face challenges like lack of persistence and antigenic epitope loss, which may result in immune escape and subsequent tumour recurrence. CAR‐T cell therapy can induce toxicities, among which the most common and severe is cytokine release syndrome (CRS) [12]. CRS is an acute inflammatory response marked by a significant elevation in pro‐inflammatory cytokines, triggered directly by T cell activation. Tumour lysis syndrome (TLS) and macrophage activation syndrome (MAS), both serious and potentially life‐threatening, are commonly observed after CAR‐T cell infusion targeting tumour cells. The ex vivo manipulation and transfer of CAR‐T cells may enhance immunogenicity due to the introduction of non‐physiological sequences, leading to immune rejection or unintended targeting of self‐tissues [76].

Notably, in the clinical setting, a correlation was observed between gp350 expression in EBV‐associated tumours and cytokine expression, suggesting that cytotoxic responses against EBV can coexist with the tumour's tolerogenic microenvironment [73]. Additionally, a potential limitation, especially in targeting solid tumours, is the efficient trafficking of engineered T cells to disease sites. Extensive research into T cell trafficking mechanisms has identified soluble factors, receptors, and adhesion molecules that play crucial roles in mediating this process [77].

And a recent Food and Drug Administration (FDA) gathered evidence and pointed out that there were increased risk of secondary T‐cell malignancies in patients after commercial CAR T‐cell therapy [78]. Further studies are required to identify potential predictors, understand the underlying mechanisms, and develop effective treatments for the toxicities associated with CAR‐T cell infusion. To overcome immune evasion, other potential EBV glycoprotein targets, combinations of different targets, or adjunctive immune therapies should be evaluated to determine whether a synergistic effect on tumour control and enhanced immune evasion can be achieved [79].

Innovative approaches aim to prolong the persistence of engineered T cells in vivo by differentiating pluripotent stem cells into LMP2‐specific CTLs, followed by the construction of LMP1‐specific CAR‐T cell vectors. The incorporation of these CARs into dual‐receptor rejuvenated cytotoxic T lymphocytes (DRrejTs) significantly enhances their ability to elicit rapid and sustained cytotoxic responses in preclinical study [80]. Additionally, researchers have developed TCR‐like CAR‐T cells that preserve epitope specificity. These constructs modulate CAR affinity to align with TCR affinity, reducing off‐target effects while maintaining therapeutic efficacy [81]. Modifying the tumour microenvironment or matching the chemokine receptors on activated T cells with the chemokines secreted by the tumour may enhance the trafficking and anti‐tumour efficacy of adoptively transferred T cells [77]. These approaches show promise in overcoming the limitations of CAR‐T therapy, while additional clinical trials are needed to evaluate both effectiveness and safety.

## 6 DC‐Based Vaccines for EBV‐Associated Cancers

Unlike engineered T cells, which directly target and eliminate tumour cells via antigen‐specific mechanisms, DC vaccines leverage antigen presentation to elicit robust immune responses, providing another promising therapeutic avenue. Notably, DC‐based therapies exhibit a favourable safety profile and good immune tolerance across multiple tumour types [82, 83].

In a Phase I clinical trial (NCT05635591), a first‐in‐class autologous DC vaccine, KSD‐101, was developed to target and control EBV‐associated haematological diseases. KSD‐101 is prepared by loading autologous DCs with lysates from EBV‐transformed B‐LCL, which encompass a broad spectrum of EBV antigens. Among nine patients with R/R EBV‐associated haematological neoplasms, four completed the 12‐week follow‐up after their first injection. Remarkably, the trial reported a 100% overall response rate and a 100% CR rate, with no grade 3 or higher toxicities observed [84].

### 6.1 LMP Antigens as Therapeutic Targets in NPC

The LMP2 antigen is regarded as an ideal target for DC‐based therapies in type II EBV‐associated malignancies. A pilot study involving 29 NPC patients treated with the LMP2‐DC vaccine demonstrated a 5‐year survival rate of 94.4% in responders, compared to 45.5% in non‐responders [85].

Autologous adenoviral vector‐transduced DCs enhance anti‐LMP2 immune responses by presenting multiple epitopes across diverse patient MHC alleles, potentially reducing the risk of immune evasion associated with single‐epitope targeting [14]. A Phase II clinical trial tested a DC vaccine transduced with an adenovirus encoding truncated LMP1 and full‐length LMP2 in patients with EBV‐positive metastatic NPC. The trial demonstrated limited efficacy, with 3 of 16 patients showing partial responses, a median progression‐free survival (PFS) of 1.92 months, and a median overall survival (OS) of 6.0 months for the cohort [86].

### 6.2 CD137 and Its Role in DC‐Based Immunotherapy

CD137 and its ligand, CD137L, belong to the type I and type II tumour necrosis factor (TNF) receptor superfamily. These molecules play vital roles in T‐cell proliferation, differentiation, and activity regulation. CD137 is predominantly expressed on the surface of activated T cells and NK cells, where it synergizes with CD28 to provide co‐stimulatory signals essential for Th1 cell development and differentiation [87]. CD137L, expressed on antigen‐presenting cells, engages with CD137 to initiate reciprocal signalling, which amplifies immune responses against pathogens and malignant cells. On the CD137 side, the interaction activates T cell proliferation and survival, as well as enhances cytokine production through the involvement of TRAFs, NF‐κB signalling, and other pathways. This process enhances the presentation of MHC‐antigen peptide complexes and stimulates the release of cytokines and chemokines, orchestrating the immune response to EBV‐infected cells or malignant tumour cells [88, 89, 90].

A Phase I clinical trial evaluated CD137L‐expressing DCs in patients with metastatic NPC who had undergone prior systemic chemotherapy. Among 12 patients, one achieved a partial response (PR), and four had stable disease (SD). Although the objective response rate was modest, the treatment extended the median PFS to 16.5 weeks and the OS to 90.5 weeks [91].

### 6.3 Challenges and Future Directions

Despite significant advances, therapeutic DC vaccines face several challenges, including the lack of efficient preparation methods, limited immunogenicity, and difficulties in commercialization. As DC account for less than 1% of PBMC, various approaches have been explored for the generation of DC vaccines. EBV peptide‐specific T‐cell lines generated by mature DCs loaded with EBV‐specific peptide epitopes demonstrated an increased frequency; however, the immune response was not apparent and HLA‐restricted. Alternative strategies for stimulating immunity against EBV‐specific antigen epitopes involve using genetically engineered DCs infected with viral vectors [14]. While this approach overcomes the limitation of HLA restriction, it introduces new challenges, such as the immunogenicity of the virus vectors used in gene therapy and the subsequent dampening of immunity against the gene product [92].

However, the immune response induced by engineered DCs infected with viral vectors still exhibits limited efficacy [86]. The suboptimal immune effects of DC vaccines may stem from immune suppression within the tumour microenvironment, as tumour cells exploit various mechanisms to evade immune surveillance and drive their progression [15]. Overcoming this immunosuppressive barrier is essential for the success of DC‐based immunotherapy. Remodelling the tumour microenvironment may reduce immune suppression and enhance tumour cell recognition by immune cells, strengthening the antiviral response [93].

One of the major barriers to the widespread use of DC‐based vaccines for various tumours is the challenges in commercialization. To date, few Phase III trials have been conducted with DC‐based immunotherapy, primarily due to the underdeveloped potential of DC vaccines and the difficulty in securing financial support. Pharmaceutical companies are often hesitant to invest in labor‐intensive, patient‐specific vaccines, given the risk of quality issues upon reaching the production site [94].

In most single‐arm DC studies, the immunological response has shown poor correlation with clinical outcomes. Other clinical trial endpoints or immunological markers, rather than solely PFS, may be more appropriate for evaluating the therapeutic efficacy of DC vaccines [95]. Future research should focus on exploring the factors that affect vaccine efficacy, including the method of DC vaccine administration, as well as the timing and frequency of vaccination, to optimise therapeutic outcomes [85, 91]. Additionally, combining DC vaccines with cytokines or other therapies, such as adoptive CTL therapy, chemotherapy, and targeted therapies, may enhance therapeutic efficacy as either a primary or adjunctive treatment [94].

## 7 Discussion

For patients with EBV‐associated diseases, conventional radiotherapy or chemotherapy remains the standard first‐line treatment, and has demonstrated relatively positive clinical outcomes. For example, radiotherapy is the primary modality for all stages of non‐metastatic NPC due to its radiosensitive nature [39]. However, these traditional treatments are frequently associated with substantial side effects, such as tissue damage and functional impairment, as well as a heightened risk of recurrence or metastasis. Additionally, treatment‐related immunosuppression may increase mortality. These limitations have prompted growing interest in immune‐based, tumour‐specific therapies that offer lower toxicity and greater precision [45]. The immune system is capable of recognising tumour‐associated antigens and eliminating EBV‐infected or malignant cells through cytotoxic lymphocyte activation—an avenue that supports the development of EBV‐targeted cellular immunotherapy, particularly for R/R EBV‐associated malignancies.

Current EBV‐specific cellular therapies primarily target malignancies such as EBV‐PTLD, NPC, and lymphoma. Among these, adoptive of EBV‐specific CTL therapy has made significant strides, now serving as a recommended second‐line treatment for R/R EBV‐PTLD and as a preventive strategy against PTLD. CTLs can traverse microvascular barriers, infiltrate tumour sites, expand upon antigen recognition, and directly kill malignant cells through multiple mechanisms. Current research aims to refine delivery strategies and scalable manufacturing to ensure efficacy and safety in viral and tumour contexts, while reducing the risk of alloreactivity [27, 33]. Trials involving LMP‐specific CTLs in combination with chemotherapy have shown promise, although the lower immunogenicity of type II latent antigens remains a challenge [8].

CAR‐ and TCR‐T cell therapies targeting EBV antigens represent emerging therapeutic directions. Genetic engineering enables T cells to recognise tumour cells via CARs or TCRs specific for EBV‐related surface molecules. Key antigens like gp350 and LMP are abundantly expressed on infected and malignant cells, making them ideal targets. In addition, adoptive transfer of ex vivo–engineered T cells helps overcome key limitations of CTL therapy—including long production timelines and donor dependency—by enabling large‐scale generation of potent, tumour‐specific effector cells [74, 75]. For HL and NHL, despite high cure rates with conventional therapies, relapsed cases pose challenges. EBV‐CTL therapy and DC vaccine show promise, offering favourable remission rates and minimal adverse effects in small clinical trials. DC‐based immunotherapy leverages the ability of DCs to activate both naïve and memory T cells, but systemic immunosuppression may limit its efficacy. Improved vaccine formulations, rational combination strategies with other cell therapies, and better patient stratification—particularly focusing on those with low tumour burden—may enhance future outcomes [84, 86].

In conclusion, the unique antigenic landscapes of EBV‐associated cancers necessitate tailored immunotherapies. CTLs, engineered T cells, and DC vaccines represent advanced therapeutic strategies are continually evolving and have shown promising efficacy in clinical trials. Nevertheless, the widespread clinical implementation of these cellular immunotherapies has yet to be realised due to several factors, including EBV‐mediated immune evasion mechanisms, the lack of standardised protocols for cell preparation and infusion, and heterogeneity in patients' immune status. Despite the low persistence of adoptively transferred T cells, cellular immunotherapy faces additional challenges. For instance, TCR‐mediated T cell activation in the absence of co‐stimulatory signals may lead to immune tolerance rather than productive immunity. Furthermore, ex vivo manipulation of T cell products designed to enhance their therapeutic potency may inadvertently increase their immunogenicity post‐infusion, potentially resulting in severe off‐target/off‐tumour toxicities and adverse events associated with secondary immune activation [76]. Future research should prioritise resolving these factors that impede therapeutic efficacy and rigorously assessing the safety of these approaches, aiming to advance their clinical application in the treatment of EBV‐associated diseases.

## Author Contributions

**Yang Gao:** conceptualization (lead), methodology (lead), project administration (equal), resources (equal), validation (lead), visualization (lead), writing – original draft (lead), writing – review and editing (equal). **Di Wang:** conceptualization (equal), methodology (equal), validation (equal), visualization (equal), writing – review and editing (equal). **Chunrui Li:** conceptualization (equal), data curation (equal), funding acquisition (lead), methodology (supporting), project administration (equal), resources (lead), writing – review and editing (lead).

## Consent

The authors have nothing to report.

## Conflicts of Interest

The authors declare no conflicts of interest.

## Data Availability

Data sharing is not applicable to this article, as no data sets were generated or analysed for this review article.
